# Constituents of the Essential Oil of *Suregada zanzibariensis* Leaves are Repellent to the Mosquito, *Anopheles gambiae* s.s.

**DOI:** 10.1673/031.010.5701

**Published:** 2010-06-09

**Authors:** Ester Innocent, Cosam C. Joseph, Nicholas K. Gikonyo, Mayunga H.H. Nkunya, Ahmed Hassanali

**Affiliations:** ^1^ Institute of Traditional Medicine, Muhimbili University of Health and Allied Sciences, P.O. Box 65001, Dar es Salaam, Tanzania; ^2^ International Centre of Insect Physiology and Ecology, P.O. Box 30772-00100, Nairobi, Kenya; ^3^ Department of Chemistry, University of Dar es Salaam, P.O. Box 35061, Dar es Salaam, Tanzania; ^4^ Department of Pharmacy & Complementary/Alternative Medicine, Kenyatta University, P.O. Box 4384400100, Nairobi, Kenya; ^5^ Current address: Department of Chemistry, Kenyatta University, P.O. Box 43844-00100, Nairobi, Kenya

**Keywords:** Methyl ketone terpenes, traditional uses of plants, Verbenaceae

## Abstract

In traditional African communities, repellent volatiles from certain plants generated by direct burning or by thermal expulsion have played an important role in protecting households against vectors of malaria and other diseases. Previous research on volatile constituents of plants has shown that some are good sources of potent mosquito repellents. In this bioprospecting initiative, the essential oil of leaves of the tree, *Suregada zanzibariensis* Verdc. (Angiospermae: Euphobiaceae) was tested against the mosquito, *Anopheles gambiae* s.s. Giles (Diptera: Culicidae) and found to be repellent. Gas chromatography (GC), GC-linked mass spectrometry (GC-MS) and, where possible, GC-co-injections with authentic compounds, led to the identification of about 34 compounds in the essential oil. About 56% of the constituents were terpenoid ketones, mostly methyl ketones. Phenylacetaldehyde (14.4%), artemisia ketone (10.1%), (1*S*)-(-)-verbenone (12.1%) and geranyl acetone (9.4%) were the main constituents. Apart from phenylacetaldehyde, repellent activities of the other main constituents were higher than that of the essential oil. The blends of the main constituents in proportions found in the essential oil were more repellent to *An. gambiae* s.s. than was the parent oil (p < 0.05), and the presence of artemisia ketone in the blend caused a significant increase in the repellency of the resulting blend. These results suggested that blends of some terpenoid ketones can serve as effective *An. gambiae* s.s. mosquito repellents.

## Introduction

In rural Africa communities, thermal expulsion and direct burning of aromatic plants before sleeping continue to play a very important role in household protection against mosquito vectors of dangerous diseases such as malaria, yellow and dengue fever, elephatiasis and lymphatic filariasis (Norbert et al. 2003; Seyoum et al. 2003). Leaves of *Suregada zanzibariensis* Verdc. (Angiospermae: Euphobiaceae) are used for this purpose by people of the coastal region of Tanzania, and they are used also in traditional medicine for treating skin diseases, asthma, abdominal pains, and malaria (Atal et al. 1978; Hedberg et al. 1983; Omulokoli et al. 1997). The plant, known as ‘mehungwa pori’ (meaning “wild citrus”) in Kiswahili language, has leaves that resemble those of citrus plants which exhibits insecticidal (Al Dakhil and Morsy 1999; Ezeonu et al. 2001) and repellent (Oshaghi et al. 2003) activities against various mosquito species. *Suregada zanzibariensis* is well distributed in tropical Africa and grows as trees of about 9 m height (Redcliffe-Smith 1987). No follow up phytochemical study and characterization of bio-active constituents of the plant has been reported. The goal of this bioprospecting initiative was to search for plants and their phytochemicals that could be useful in mosquito control. Towards this end, the constituents of the essential oil of the plant were characterized and were studied, individually and in blends, for their repellency against *Anopheles gambiae* s.s. Giles (Diptera: Culicidae).

## Materials and Methods

### Plant materials and extraction

Leaves of *S. zanzibariensis* were collected from Pugu Forest Reserve in Pwani region, Tanzania. The plant materials were authenticated at the Herbarium of the Department of Botany, University of Dares-Salaam, where voucher specimens were deposited. The leaves were dried in the shade for 4–6 days and then hydro-distilled using a Clevenger-type apparatus for 8 h. The distilled oil was separated from the aqueous layer, dried over anhydrous Na2SO4, and stored at 4° C.

### Gas chromatography and gas chromatography-mass spectrometry

Analysis of the essential oil was carried out on a Hewlett Packard 5890A gas Chromatograph (GC) equipped with a flame ionization detector and a Hewlett Packard 3396 series II integrator. A cross-linked methyl silicon capillary column (50 m × 0.2 mm id × 0.33 µm film thickness) was used for separation of the essential oil components. Nitrogen was used as the carrier gas at a flow rate of 0.84 ml/min. The injector and detector temperatures were maintained at 250° C and 270° C respectively. The temperature program consisted of an initial temperature of 40° C, which was raised at a rate of 10° C/min to 140° C where it was maintained for 15 min. The temperature was then increased 10° C/min to 180°C and maintained for 15 min, and then increased at 10° C/min to 280° C and maintained for 15 min. Identification of the essential oil components was carried out on a Hewlett Packard 5790A series GC coupled to the VG Masslab 12–250 mass spectrometer (Micromass, Waters Inc., www.waters.com) with mass range *m*/*z* 1-1400. The spectrometer was equipped with a computerized data system running using MassLynx (Waters) software with Wiley Version 6 and NIST Version 1.0 MS libraries. The spectrometer was operated in the EI mode at 70 eV with temperature of the source held at 180° C, multiplier voltage at 1350V, scan cycle of 1.5 s (scan duration of 1 s and inter-scan delay of 0.5 s), and scan range of *m/z* 38–650. The instrument was calibrated using heptacosafluorotributyl amine, [CF3(CF2)3]3N, (Apollo Scientific Ltd., www.apolloscientific.co.uk). The column and temperature program used for GC-MS was the same as for GC analysis except for the carrier gas, which was helium in this case. Where possible (depending on availability), identities of the essential oil components were confirmed by GC coinjections with authentic samples obtained from Sigma Aldrich Chemical Co. (www.sigmaaldrich.com) or Fluka (Sigma). Identification of the other compounds that were not commercially available was based on detailed comparison of their mass spectra with those in the libraries.

### Mosquitoes

Mated female adult *An. gambiae* s.s. mosquitoes used in the study were obtained from a colony reared according to World Health Organization (WHO) (1996) protocol at International Centre of Insect Physiology and Ecology insectary (initially cultured from specimens obtained from Ifakara in Tanzania in 1998). The larvae were reared at 32–36° C on a diet of TetraMin® (Tetra GmbH, www.tetra.de). The adult mosquitoes were maintained in an insectary at 26–28° C and 70–80%, RH. on a 6% glucose solution. Females fed on humans three times per week. Female mosquitoes used in the experiments were 5–7 days old, initially maintained on human blood but changed to glucose (6% solution) a day before the bioassays and then starved for 18 h before use.

### Ethical clearance and volunteer safety

As the experiment required sources of human blood for mosquitoes and human landing catches, local volunteers were recruited with informed consent. A research protocol was submitted to the International Centre of Insect Physiology and Ecology, based at Duduvile-Nairobi, and to the Kenya National Ethical Review Committees, based at the Kenya Medical Research Institute. Ethical clearance was obtained from the Kenya National Ethics Board. The discomfort and potential risks of mosquito bites was explained to the volunteers. The individuals had previously participated in similar studies, had good knowledge of malaria transmission, and showed mild or no allergic reaction to mosquito bites or the essential oil. Five adult volunteers (3 male and 2 female) were involved in the experiments, and they did not object to being identified for publication. A parasite-free environment was ensured through regular screening of the volunteers' peripheral blood for *Plasmodium*. Sulphadine-pyriproxyfen prophylaxis was provided to each volunteer.

### Repellency assays

The repellency assay was performed in a dark room with red light as the only source of illumination (WHO 1996). The room temperature and humidity were controlled at 28 ± 2° C and 75 ± 5% RH respectively to mimic the feeding conditions for female *An. gambiae* s.s. mosquitoes. Cages (50 × 50 × 50 cm) made of aluminium sheet at the bottom, Pyrex window screen on sides and top, and a cotton stockinet sleeve for access on the front, were used in the dose response assays. Different concentrations (0.01–10%) w/v) of the essential oil, selected constituents, and blends of these were prepared by dissolving 1 g of each sample in 10 ml of analytical grade acetone (99.95%)), followed by successive ten-fold dilutions with acetone to obtain the other concentrations. (1*S*)-(-)-verbenone (17) and other compounds whose repellency against *An. gambiae* had previously been reported (Omolo et al. 2004, 2005; Odalo et al. 2005) were not evaluated singly, but as constituents of blends. The blend of the four main constituents of the oil (phenylacetaldehyde (5), verbenone (17), artemisia ketone (11), and geranyl acetone (24)) in relative amounts present in the essential oil (14:12:10:9 gram ratios), as well as four blends with one of each of these constituents missing, were prepared. Acetone acted as a blank in all experiments, and DEET acted as a positive control. Fifty test mosquitoes were used in each of five replicates involving five different adult volunteers for each concentration of a sample. The volunteers had no contact with any lotion, perfume, oil, or perfumed soap on the day of the bioassay. The forearm (average area of 696.6 cm2) of each volunteer from the elbow to the hand was washed with water and left to dry. The test sample (1 ml) was spread as evenly as possible on one of the forearms of a volunteer from the wrist to the elbow. The rest of the hand was covered with a glove. Acetone (1.0 ml) was dispensed on the other forearm to serve as control. The control and treated arms were interchanged regularly to eliminate bias. The control arm was first introduced into the cage for 3 min immediately after introduction of the mosquitoes. The number of mosquitoes that landed on the arm was recorded, and the insects were shaken off before they imbibed any blood. This was followed by exposure of a volunteer arm first to the lowest concentration (0.01% w/v) of the test sample followed by sequential exposures to progressively higher concentrations (0.1, 1, 10% w/v) of the sample, each time to fresh mosquitoes in a clean cage. The test arm of the volunteer was washed using a non-perfumed soap and tap water and allowed to dry naturally for at least 20 min before dispensing the subsequent concentration. Only one compound/sample was tested per day.

### Data analysis

Percentage protective efficacy (PE) was calculated using the formula PE = (C-T/C) × 100%, where C and T are the mean numbers of mosquitoes that landed on the control and test arm, respectively (Sharm and Ansari 1994; Matsuda et al 1997). Means were subjected to analysis of variance (ANOVA) and compared by the Student-Newman-Keuls test (SAS 2000). Probit to compute repellency concentration that caused 50% response of the test mosquitoes (RC50) was done using the Lackfit inversel of the SAS programme (SAS 2000).

## Results and Discussion

### Chemical composition of essential oil from *S. zanzibariensis*

**Figure 1.**
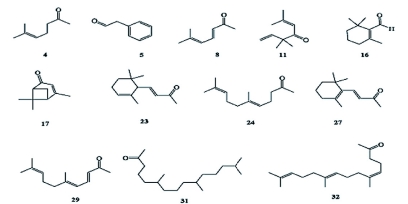
Chemical structures of methyl ketone terpenes and main constituents of *Suregada zanzibariensis* essential oil. High quality figures are available online.

The yield of essential oil from the dried leaves of *S. zanzibariensis* was 0.004%. Table 1 shows the composition of the essential oil and the compounds (representing ∼80% of all constituents of the essential oil) that were identified. Phenylacetaldehyde (5) (14.4%), artemisia ketone (11) (10.1%), (1*S*)-(-)-verbenone (17) (12.1%), and geranyl acetone (24) (9.4%) were the main constituents. Ketone compounds accounted for 56.5% of all identified components. Methyl ketone terpenes included 6-methyl-5-hepten-2-one (4), *cis*-6-methyl-3,5-heptadien-2-one (8), α-ionone (23), geranyl acetone (24), β-ionone (27), pseudoionone (29), 6, 10, 14-trimethyl pentadecan-2-one (31) and farnesyl acetone (32) (Figure 1).

**Table 1.**
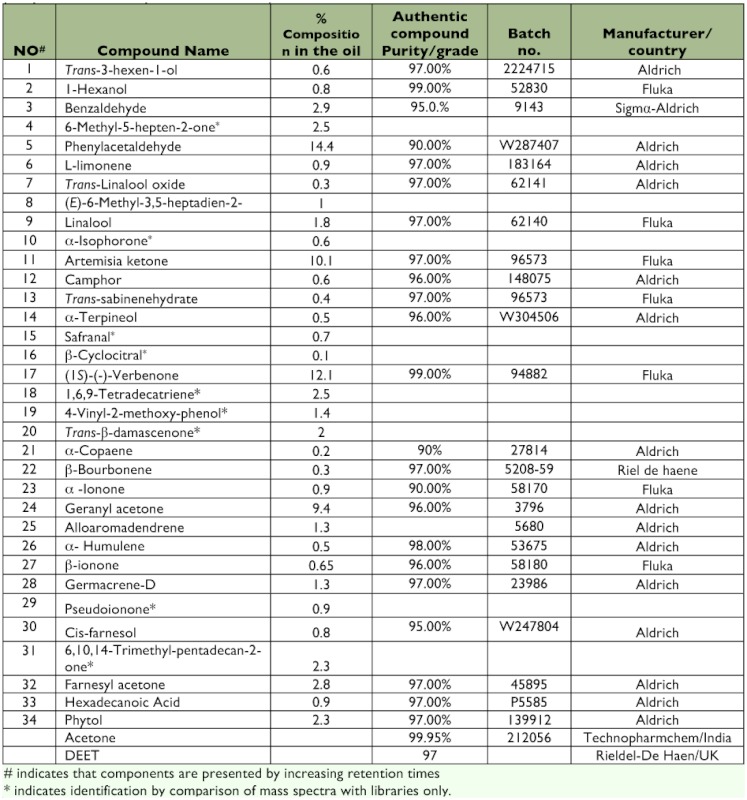
Percentage composition of compounds detected in the essential oil from *Suregada zanzibariensis* and percentage purity of authentic compounds used in co-injections.

### Repellency activity of *S. zanzibariensis* essential oil, compounds and blends

Among the compounds tested in this study, methyl ketone terpenes exhibited good repellency against *An. gambiae* s.s. (Table 2), which is consistent with previous studies that reported that aliphatic methyl ketones exhibit good mosquito repellency activities (Barton 2003; Roe 2004; Innocent 2008). Repellency activity of geranyl acetone, β-ionone, and farnesyl acetone (Table 2) compared well with other plant-based mosquito repellents (Dethier 1947; Grayson 2000). Repellency activity of β-ionone (27) was higher than its acyclic counterpart geranyl acetone (24). Likewise, farnesyl acetone (32) was more repellent than geranyl acetone (24). 6-Methyl-5-hepten-2-one (4), which has been identified as a defensive allomone of many insects such as ants, termites, and cockroaches (Blum 1996), was not commercially available and could not be assayed in this study. Interestingly, 6-Methyl-5-hepten-2-one (4) and geranyl acetone (24) previously identified in the headspace of fresh and incubated sweats of humans, were shown to elicit response to the neurons innervating a grooved peg sensillum of a female *An. gambiae*, and were electro-attennogram active (Meijerink et al. 2000, 2001).

**Table 2:**
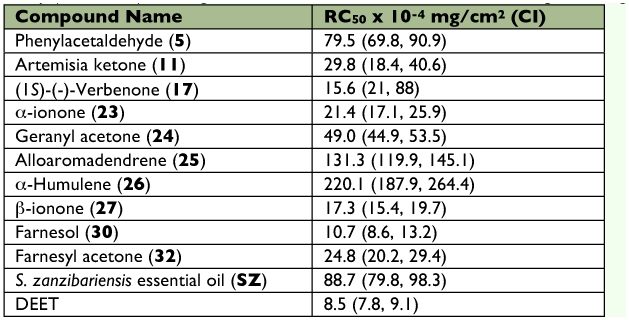
Repellency activity (RC Values) of *Suregada zanzibariensis* essential oil constituents against *An. gambiae s.s.*

**Figure 2.**
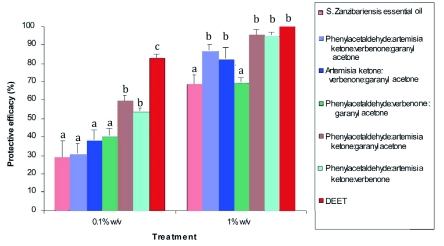
Percentage protective efficacy (± SE) of *Suregada zanzibariensis* essential oil and blends of the main constituents against *Anopheles gambiae* s.s. Ratios of weight of the main constituents used in preparation of blends are 14:12:10:9 for phenylacetaldehyde (5): verbenone (17): artemisia ketone (11): geranyl acetone (24); Columns with the same letters at a given concentration are not significantly different at P < 0.05 (Student-Newman-Keuls test). High quality figures are available online.

The repellent activity of constituents in the oil was dose dependent (p < 0.05). At concentration of 1% w/v, the blend of the four major constituents in the proportion found in *S. zanzibariensis* essential oil was higher than that of the crude essential oil (p < 0.05) indicating additive or synergistic effects of the compounds. The presence of artemisia ketone (11) caused a significant increase in the activity of the blends (Figure 2). Although none of the assayed constituents exhibited repellency comparable to that of DEET, the repellency of some of the blends at 1% w/v was comparable to that of this synthetic repellent, suggesting that further screening of different blends may lead to the discovery of more repellent combinations.

## Abbreviations

GC: gas chromatography;
MS: mass spectrometry;
